# Vaccine Response in the Immunocompromised Patient with Focus on Cellular Immunity

**DOI:** 10.3390/vaccines10060882

**Published:** 2022-05-31

**Authors:** Christina Bahrs, Nicole Harrison

**Affiliations:** 1 Institute for Infectious Diseases and Infection Control, Jena University Hospital—Friedrich Schiller University, 07747 Jena, Germany; 2 Department of Medicine I, Division of Infectious Diseases and Tropical Medicine, Medical University of Vienna, 1090 Vienna, Austria; nicole.harrison@meduniwien.ac.at

During the last few years, we have experienced a shift in how we evaluate the effectiveness of vaccines. For decades, the measurement of specific antibodies produced in response to vaccination was the surrogate marker for protection against vaccine-preventable diseases. While clinicians still rely on the humoral response when assessing responses to a vaccine, the cellular immune response has been attracting an increasing amount of attention in recent years, most prominently since the SARS-CoV-2 pandemic [1,2,3]. In immunocompromised patients, especially those who are severely immunosuppressed from procedures such as stem cell transplantation or solid organ transplantation with life-long immunosuppression, the antibody response to vaccination is usually strongly diminished [4,5,6]. However, these patient groups are most vulnerable to infections [7,8], and therefore, improving vaccination strategies is of utmost importance. The following articles shed new light on some aspects of the cellular immune response to vaccination in immunocompromised patients.

In this Special Issue, Rüthrich and colleagues discuss the known evidence regarding the cellular immune response, in particular the T-cell response, to different types of vaccines in cancer patients [1]. In this extensive review, the authors emphasize the fact that various clinical studies have already shown that the antigen-specific T-cell response after vaccination is more robustly and reliably induced in this vulnerable population than the antibody response [9,10,11]. Moreover, Rüthrich and colleagues underline the fact that compared to the humoral vaccine response, T cells are more likely to be cross-reactive to influenza strains or SARS-CoV-2 variants that differ from the vaccination strains or strains that caused a prior infection [1,12,13]. Thus, the authors conclude that in particular, T-cell vaccine-induced immune responses might be reliable markers for protection, which should be considered in the development of novel vaccines, especially for patients who have impaired immune responses [1].

Additionally, another important aspect, the challenge of adapting immunization schedules and/or basic therapy for patients with haematological malignancies, is highlighted in a case report by Kratzer and colleagues [14]. A 58-year-old patient who suffered from multiple myeloma achieved complete remission after autologous stem cell transplantation and then received long-term maintenance treatment with the second-generation immunomodulatory agent pomalidomide for three years. After basic immunization with the mRNA vaccine BNT162b2, the authors did not detect any signs of seroconversion or T-cell-specific memory. To improve the SARS-CoV-2 specific vaccine response, the attending physicians administered heterologous SARS-CoV-2 vaccination with two additional vaccinations with the vector-based vaccine ChAdOx1 and stopped the treatment with pomalidomide. Thereafter, the patient seroconverted with moderate spike-protein-specific antibody levels reaching 49 BAU/mL and achieved SARS-CoV-2-specific T-cell responses (T-cell proliferation, effector cytokine production (interleukin 2 and interleukin 13) and T-cell activation with increased numbers of CD3^+^CD4^+^CD25^+^ T cells) [14]. However, despite repeated heterologous SARS-CoV-2 vaccination, the patient did not develop neutralizing receptor-binding domain-specific antibodies. Therefore, despite the immunomonitoring-based adjustment of the vaccination and therapy schedule, this patient can only be regarded as poor responder and might be a candidate for passive SARS-CoV-2 immunization.

The establishment of feasible diagnostic methods to assess the cellular immune response is the first step in integrating this approach in the evaluation of vaccine response in immunocompromised patients. In this Special Issue, Gäckler and colleagues developed an interferon-gamma ELISPOT assay sensitive enough to detect vaccine-induced T-cell responses against *Streptococcus pneumoniae* in 38 clinically stable kidney transplant recipients who received sequential vaccination with the 13-valent pneumococcal conjugate vaccine followed by the 23-valent pneumococcal polysaccharide vaccine [15]. This is the first study that was able to show an increase in serotype-specific cellular immunity after pneumococcal vaccination in organ transplant recipients. The authors observed the strongest cellular immune responses against pneumococcal serotypes 9N and 14. The interferon-gamma ELISPOT assay established by Gäckler and colleagues [15] is a technique which could be used for further studies in this area involving larger immunocompromised patient cohorts.

Patients who have undergone allogeneic hematopoietic stem cell transplantation (HSCT) belong to the most immunosuppressed patient populations, with several factors such as a lack of immune reconstitution and graft-versus-host-disease which can severely diminish the response to vaccination [6,16]. In this Special Issue, Lindemann and colleagues assessed the humoral and cellular vaccination response in 117 HSCT recipients vaccinated with two doses of a SARS-CoV-2 vaccine (mainly the m-RNA vaccine BNT162b2) compared to 35 vaccinated healthy volunteers [17]. T-cell immune response was assessed using an in-house interferon-gamma ELISPOT and the commercially available T-SPOT.COVID using seven SARS-CoV-2-specific antigens altogether. After two vaccinations, HSCT patients had significantly lower SARS-CoV-2-specific antibody levels compared to healthy controls (*p* < 0.001). Additionally, T-cell-mediated immune responses were significantly reduced to ≤33% compared to healthy controls, depending on the spike antigen employed for in vitro T-cell stimulation. Interestingly, gender had a significant impact on antibody responses after vaccination. Female HSCT patients showed significantly higher antibody ratios compared to male HSCT patients, whereas T-cell responses were comparable between male and female HSCT patients [17]. This study highlights the need for adapted vaccination schedules/compositions or passive immunization with monoclonal antibodies to meet the needs of severely immunosuppressed patients. Meanwhile, the European Medicines Agency recommends four doses of mRNA vaccines in immunocompromised individuals who experience a suboptimal response to earlier vaccination [18]. However, there are currently no data available on immunogenicity, safety or effectiveness in this population [18].

In another single-centre prospective study from Vienna, Austria, an area that is endemic for tick-borne encephalitis (TBE) virus with high vaccination coverage of the population [19], the authors focused on the cellular response after TBE vaccination in allogeneic HSCT patients compared to unvaccinated healthy controls [20]. This study showed that all HSCT patients with a significant humoral response to vaccination (at least 2-fold increase in neutralization titres after two doses of the TBE vaccine) [5] already exhibited strong TBE-specific lymphocyte proliferative responses measured using a thymidine incorporation assay (stimulation index > 10) prior to TBE vaccination after HSCT [20]. Moreover, HSCT patients with vaccinated sibling donors were more likely to elicit a strong TBE-specific lymphoproliferative and cytokine response (mainly by the Th2 cytokine interleukin-13) compared to patients with unrelated donors of unknown vaccination status [20], suggesting that TBE-specific memory T cells were derived from the related donors. Therefore, the vaccination of donors might play an important role in the transfer of cellular immunity to HSCT patients, and a booster vaccination could even be offered to related donors prior to stem cell donation, which might improve the vaccine response in HSCT recipients post-transplant.

Overall, this Special Issue provides new insights into cellular immunity, in particular antigen-specific T-cell and cytokine responses to different vaccines in severely immunocompromised patients. In future, better and more feasible techniques are necessary to assess the cellular response after vaccination and guide the vaccination schedule, composition and basic therapy of immunosuppressed patients. In special settings, the passive immunization of patients or the booster vaccination of stem cell donors might also be of value to this vulnerable population.

## Data Availability

Not applicable.
